# Acute interstitial nephritis associated with ingesting a *Momordica charantia* extract: A case report

**DOI:** 10.1097/MD.0000000000026606

**Published:** 2021-07-09

**Authors:** Wooram Bae, Seongmin Kim, Jungyoon Choi, Tae Won Lee, Eunjin Bae, Ha Nee Jang, Sehyun Jung, Seunghye Lee, Se-Ho Chang, Dong Jun Park

**Affiliations:** a Department of Internal Medicine, Gyeongsang National University Changwon Hospital, Changwon, South Korea; b Department of Internal Medicine, Gyeongsang National University College of Medicine, South Korea; c Institute of Health Science, Gyeongsang National University, Jinju, South Korea; d Department of Internal Medicine, Gyeongsang National University Hospital, Jinju, South Korea.

**Keywords:** drugs, *Momordica charantia*, nephritis, nutraceuticals, side effects

## Abstract

**Rationale:**

*Momordica charantia* is often used to treat type 2 diabetes mellitus in Korea. Drug-induced acute interstitial nephritis (AIN) accounts for 60% to 70% of AIN cases. However, only 1 case of AIN associated with ingesting *M charantia* has been reported in the English literature. We report an extremely rare case of AIN that occurred after a patient ingested a pure *M charantia* extract over 7 months.

**Patient concerns:**

A 60-year-old Korean woman was admitted to our hospital for a renal biopsy. Her renal function had decreased gradually over the last 9 months without symptoms or signs.

**Diagnosis:**

Her blood urea nitrogen and serum creatinine levels were 29.7 mg/dL (range: 8.0–20.0 mg/dL) and 1.45 mg/dL (range: 0.51–0.95 mg/dL) on admission. Renal histology indicated AIN; there was immune cell infiltration into the interstitium, tubulitis, and epithelial casts, although the glomeruli were largely intact.

**Interventions:**

*M charantia* was discontinued and prednisolone was prescribed.

**Outcomes:**

The value of serum creatinine has almost been restored to the baseline level after 3 months.

**Conclusion:**

s: This is the first case report of AIN associated with the ingestion of a pure *M charantia* extract. Recognition of the possible adverse effects of these agents by physicians is very important for early diagnosis and appropriate management.

## 1 Introduction

*Momordica charantia* (family Cucurbitaceae), commonly known as bitter melon, has been used as an alternative therapy for type 2 diabetes mellitus (T2DM), hypertension, cancer, and bacterial and viral infections due to its many bioactive compounds.^[1–3]^ In particular, *M charantia* has a long history as a hypoglycemic agent in Asia, Africa, and Latin America. The extract of *M charantia* is often referred to as vegetable insulin.^[4]^ It has been widely demonstrated in cell line and animal models that *M charantia* extracts lower glycemia in T2DM patients.^[3,5–7]^ Small non-randomized and double-blind clinical trials have reported that bitter melon juice, fruit, and dried powder have a moderate hypoglycemic effect.^[4,8]^ However, clinical efficacy and safety have not been established.

Acute interstitial nephritis (AIN) is a common cause of acute kidney injury (AKI). AIN typically occurs in response to prescribed or over-the-counter drugs (60%–70% of cases).^[9–11]^ Various drugs can cause AIN, the clinical characteristics and laboratory findings of which vary by drug class. ^[11]^ AIN can be definitively diagnosed by renal biopsy. Histological findings of AIN typically include interstitial inflammation, edema, and tubulitis. Alternative medicines have been widely used in Korea despite a lack of safety data. To the best of our knowledge, only 1 case of AIN associated with ingesting *M charantia* has been reported in the English literature^[12]^ We report an additional case of AIN occurring after ingestion of pure *M charantia* extract.

## 2 Ethical statement and consent

Written informed consent was obtained from the patient for publication of their case report and any accompanying images. The study protocol was approved by the Institutional Review Board of Gyeongsang National University Changwon Hospital (IRB No. 2020-04-007).

## 3 Case report

**Figure 1:**
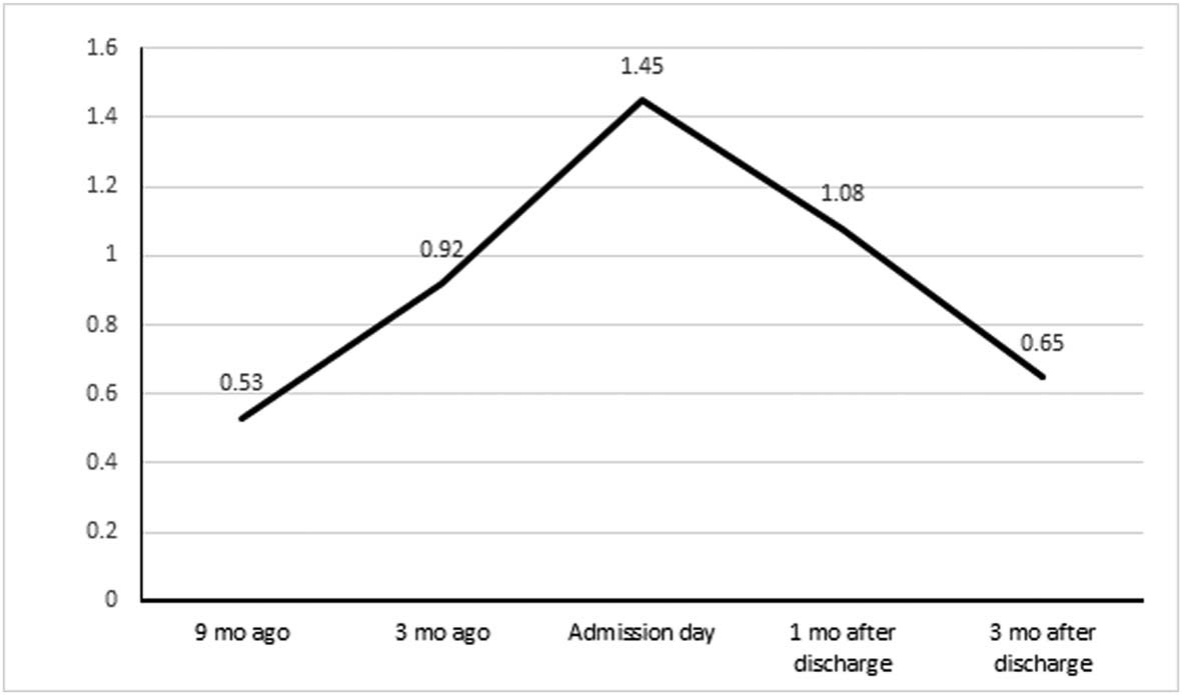
Serial change of serum creatinine before and after admission.

A 60-year-old Korean woman was admitted to our hospital for a renal biopsy. She had presented to the nephrology outpatient department due to edema 9 months earlier. She was diagnosed with T2DM and hypertension 5 years ago and took 4 mg glimepiride, 1000 mg metformin, 50 mg gemigliptin, 15 mg pioglitazone, 5 mg amlodipine, 100 mg losartan, and 25 mg carvedilol. The pioglitazone was withdrawn, while the other medicines were maintained, after the initial visit to our hospital. Her serum creatinine level had been gradually increasing (Fig. 1) without significant changes in other laboratory findings. She denied symptoms and signs indicating deterioration of renal function, such as edema, oliguria, fever, chills, general weakness, malaise, arthralgia, myalgia, skin rash, and urine color changes during the 9-month follow-up. She also denied ingestion of known toxins, Chinese herbal medicines, drugs (including non-steroidal anti-inflammatory drugs), and nutraceuticals that can affect the serum creatinine level. Her hypertension and serum glucose were well controlled. She was admitted for renal biopsy and management of AKI.

Her initial vital signs were as follows: blood pressure, 120/80 mmHg; heart rate, 68 beats/min; respiratory rate, 18 breaths/min; and body temperature, 36.5°C. No lymph nodes were palpated on a neck examination. Heart sounds were normal and no wheezing or rales was heard in either lung field. No organomegaly was present in the abdomen, and bowel sounds were audible. No skin color changes on the torso or pitting edema were observed on the legs. Her blood urea nitrogen and serum creatinine levels were 29.7 mg/dL (range: 8.0–20.0 mg/dL) and 1.45 mg/dL (range: 0.51–0.95 mg/dL) on admission, whereas they were 14.1 and 0.53 mg/dL, respectively, 9 months before admission (Fig. 1). The hematocrit and hemoglobin levels were 34% (range: 36%–48%) and 11.4 g/dL (range: 12–16 g/dL), respectively. Platelet and red blood cell counts were 232 × 10^9^/L (normal range: 130–400 × 10^9^/L) and 3.82 × 10^12^/L (normal range: 4.0–5.40 × 10^12^/L), respectively. The white blood cell count was 6.76 × 10^9^/L (normal range: 4.0–10.0 × 10^9^/L), with 63.5% segmented neutrophils (normal range: 50%–75%), 27.5% lymphocytes (normal range: 20%–44%), 6.7% monocytes (normal range: 50%–75%), and 1.9% eosinophils (normal range: 1.0%–5.0%). Other laboratory test results included sodium, 138 mmol/L (range: 135–145 mmol/L); potassium, 4.6 mmol/L (range: 3.3–5.1 mmol/L); chloride, 104 mmol/L (range: 98–110 mmol/L); glucose, 177 mg/dL (range: 70–110 mg/dL); HbA1c, 6.8% (range: 4.2%–5.9%); calcium, 9.0 mg/dL (range: 8.6–10.2 mg/dL; phosphorus, 3.9 mg/dL (range: 2.7–4.5 mg/dL); and total CO_2_, 28 mmol/L (range: 21–31 mmol/L). The C3 and C4 levels were 129.7 mg/dL (range: 90–180 mg/dL) and 34.8 mg/dL (range: 10–40 mg/dL), respectively. Urinalysis (dipstick test) revealed no protein or blood, and no red blood cells or white blood cells. The urine albumin creatinine ratio was 14.9 mg/g (range: 0–20 mg/g). The thyroid function test was within normal limits, and anti-neutrophil cytoplasmic antibodies and the anti-glomerular basement membrane antibody were negative.

**Figure 2:**
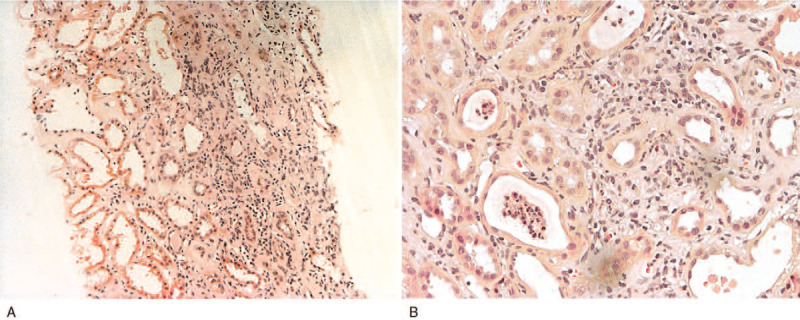
Histologic findings of renal biopsy. There was edematous interstitium, detachment of tubular epithelial cells, and lymphoplasmacytic infiltration into interstitium (A) (×100, H&E staining). Intratubular aggregates of inflammatory cells including neutrophils and cell debris (arrow). A few eosinophils (circle) present among the interstitial inflammatory cell infiltrates (B) (×200, H&E staining).

Kidney ultrasound revealed normal-sized kidneys (right, 10.4 cm; left, 10.4 cm) and echogenicity without evidence of hydronephrosis or nephrolithiasis. A renal biopsy was performed on day 2 of admission. There was edematous interstitium, detachment of tubular epithelial cells, and lymphoplasmacytic infiltration into the interstitium (Fig. 2A). Tubulitis was indicated by tubular cell detachment and epithelial casts including numerous neutrophils were observed in the lumen of renal tubules and a few eosinophils were also observed among the increased interstitial inflammatory cell infiltrates (Fig. 2B). However, the glomeruli were normal (Fig. 2). After the renal biopsy, we again enquired regarding the consumption of drugs, Chinese herbal medicines, and nutraceuticals that could evoke AIN on day 3 of admission. She disclosed that she had been ingesting an *M charantia* extract for 7 months to control her T2DM. She took about 600 mg almost every day in liquid form during the first 3 months, and had taken 1200 mg per day during the last 4 months. The *M charantia* extract was immediately withdrawn and 0.5 mg/kg/day of prednisolone was prescribed, and tapered, and then stopped after 3 months. Her serum creatinine decreased to 0.65 mg/dL after 3 months (Fig. 1), while still using the other medicines.

## 4 Discussion

A case of AIN induced by ingesting an *M charantia* extract, a nutraceutical commonly used to treat T2DM in South Korea, has been described. A thorough medical history, and periodic follow-up visits to assess renal function in the absence of clinical symptoms, leading to a definitive diagnosis in this patient. This report is significant because long-term use of a pure *M charantia* extract could result in AIN in patients with T2DM and hypertension.

Only 1 case of AIN associated with *M charantia* has been reported in a patient with T2DM and hypertension in the English literature^[12]^ That patient had taken 1 hyponidd tablet per day for 7 days before the onset of symptoms. Hyponidd usually contains 10 active ingredients, including *M charantia*. Among the various ingredients in hyponidd, *M charantia and Gymnema sylvestre* have been shown to induce nephrotoxicity in animal studies*. G sylvestre* is also commonly used to manage T2DM in ayurvedic practice. Unlike our case, renal function was sufficiently impaired in the literature case to require dialysis; the patient had still not fully recovered 2 months after admission. These differences in clinical outcomes may be due to differences in the doses of *M charantia* used, and to the effects of other ingredients, particularly *G. sylvestre,* rather than *M charantia.* Our case can be considered the first report of AIN caused solely by ingesting a *M charantia* extract.

Some in vivo experimental studies on the nephrotoxicity of *M charantia* seeds have been reported.^[13,14]^ Polypeptide-k (1000 mg/kg) isolated from *M charantia* does not have any effect on the function or histology of rat kidneys after 72 hours. In another study, 4000 mg/kg of *M charantia* extract, as a single dose, had no significant adverse effects on renal function or structure, but chronic administration of 500 mg/kg daily for 7 days resulted in a significant change in renal function and tubular damage. *G sylvestre*, another anti-diabetic nutraceutical, promotes cellular infiltration, degenerative changes, and necrosis of the tubular epithelium in rat kidneys. It was proposed that tubular injury was a direct effect of drug toxicity and an immunologically mediated indirect injury.^[15]^ In our case, renal pathology also revealed tubular damage and interstitial cellular infiltration.

Other medicines that can evoke AIN was ruled out as the cause in our case, because glimepiride, gemigliptin, amlodipine, losartan, and carvedilol, used to control her T2DM and hypertension, were taken over several years and maintained during the treatment. No recurrence of AIN has been observed despite additional metformin use after recovery of AKI. There are 2 methods for determining the etiology of AIN: the Naranjo probability scale^[16]^ and the World Health Organization-Uppsala Monitoring Center criteria.^[17]^ Our patient's Naranjo probability scale score was 6 (probable causal relationship) and the World Health Organization-Uppsala Monitoring Center classification was “probable.”

The clinical presentation of AIN varies according to the class of the drug/s involved, and the clinical course may be characterized by a wide range of symptoms and signs.^[11]^ The only consistent clinical manifestation is acute or subacute kidney injury, which often results in chronic kidney disease if improperly managed.^[18,19]^ AKI is usually non oliguric, with gradual increases in the serum creatinine level, whereas patients with severe AKI present with oliguria and show a rapidly progressive course. Therefore, a diagnosis of drug-induced AIN is often considered when unexplained renal insufficiency is detected and diagnosed after renal biopsy. Our patient did not complain of any symptoms or signs and showed no changes in any other laboratory markers except for gradual increases in blood urea nitrogen and serum creatinine. Initially, there was doubt regarding the diagnosis of *M charantia-*associated AIN because of the extreme rareness of this condition, but renal biopsy confirmed the association.

## 5 Conclusions

Alternative medicines, including nutraceuticals and herbal medicines, are used concurrently with therapeutic drugs in patients with underlying chronic diseases, and alone even in healthy persons due to the mistaken belief that there are no side effects. Physicians should enquire about *M charantia* ingestion when encountering a patient with an unexplained decrease in renal function because such patients commonly fail to disclose that they have taken an *M charantia* preparation for disease control.

## Author contributions

**Conceptualization:** Wooram Bae, Seongmin Kim, Jungyoon Choi.

**Data curation:** Wooram Bae, Tae Won Lee, Eunjin Bae.

**Formal analysis:** Ha Nee Jang, Sehyun Jung, Seunghye Lee, Se-Ho Chang.

**Investigation:** Wooram Bae, Seongmin Kim, Jungyoon Choi.

**Methodology:** Wooram Bae, Tae Won Lee, Eunjin Bae.

**Supervision:** Se-Ho Chang, Dong Jun Park.

**Validation:** Tae Won Lee, Eunjin Bae.

**Writing – original draft:** Wooram Bae.

**Writing – review & editing:** Tae Won Lee, Eunjin Bae, Dong Jun Park.
